# Mapping uncovered a multi-phase arc–back-arc system in the southern Beishan during the Permian

**DOI:** 10.1093/nsr/nwac204

**Published:** 2022-09-24

**Authors:** Tong Hong, Gabriel S Santos, Cees R van Staal, Wenhua Ji, Shoufa Lin

**Affiliations:** Department of Earth and Environmental Sciences, University of Waterloo, Canada; Department of Earth and Environmental Sciences, University of Waterloo, Canada; Department of Earth and Environmental Sciences, University of Waterloo, Canada; Geological Survey of Canada, Canada; China Geological Survey, Xi’an Centre, China; Department of Earth and Environmental Sciences, University of Waterloo, Canada; School of Resources and Environmental Engineering, Hefei University of Technology, China

The Central Asian orogenic belt (CAOB), surrounded by the Baltica and Siberia cratons to the north, and the Tarim and North China cratons to the south, records successive evolution cycles of subduction and accretion/collision from Neoproterozoic to the Paleozoic that consumed and eventually closed the Paleo-Asian Ocean [1]. Tectonic models of the terminal orogenesis are contentious, with discussions ranging from the time and location of final closure of the Paleo-Asian Ocean to the regional context and setting of its various tectonic constituents. Results of detailed mapping in the southern Beishan Orogen in the southern CAOB, conducted as part of a collaboration among China Geological Survey, the University of Waterloo (Canada) and the Geological Survey of Canada, support that parts of the Paleo-Asian Ocean were not closed until the Permian and indicate that its final closure involved accretion of several distinct terranes, including multiple Permian arc and arc–back-arc systems—a process more comprehensive than previously proposed.

The Beishan Orogen is an E–W trending collage of accreted terranes and sits between two southern suture zones of the CAOB: the Tianshan suture in the west and the Solonker suture in the east (Fig. 1a) [2]. It contains some of the youngest, Permian ophiolitic and arc complexes, and sedimentary basins in the CAOB (Fig. 1b) [3], making it a key area for investigating the contentious, terminal collision history of this orogen. Through detailed mapping (1:25 000 scale), four distinct tectono-stratigraphic packages were identified in southern Beishan: the Baidunzi Complex, the Ganquan Complex, the Liuyuan Complex and the Heishankou sedimentary sequence (Fig. 1c and d).

**Figure 1.**
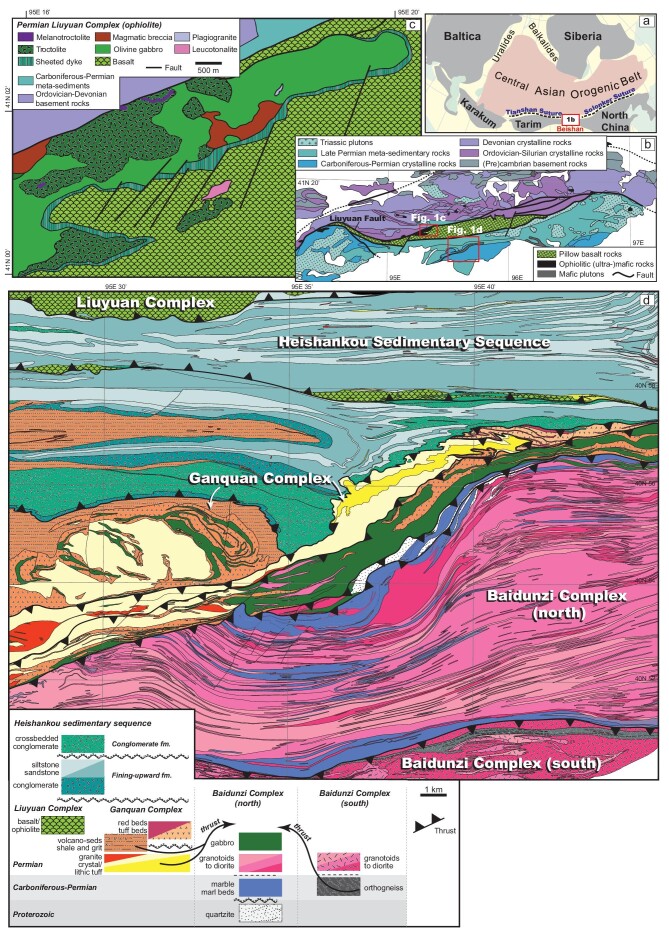
(a) Sketch of the Central Asian orogenic belt showing the locations of sutures and the southern Beishan orogen (modified from Ref.[1]). (b) Simplified tectonic map of the southern Beishan orogen (modified from Ref. [4]). (c) Simplified geological map of central part of the Liuyuan Complex (simplified from an unpublished 1:25 000 geological map). (d) Simplified geological map of the Baidunzi area (simplified from the 1:25 000 geological map [5).

The Baidunzi Complex comprises a mixture of variably sheared and isoclinally folded metasedimentary and syn-kinematic meta-plutonic rocks. The oldest known rocks in the Baidunzi Complex are thick-bedded orthoquartzite and minor argillaceous chlorite–muscovite schist. Detrital zircon analyses of the quartzite show no age peaks younger than ∼1060 Ma. The structurally overlying metasedimentary rocks include a regionally extensive unit of marble and marl associated and interlayered with quartz-rich meta-psammite and schist, indicating a shallow shelf environment. The calcareous rocks are younger than ∼297 Ma, based on their youngest detrital zircon age peak. Combined with the emplacement ages of the meta-plutonic rocks, which cut the marble, the latter were deposited between ∼297 and 294 Ma.

The meta-plutonic rocks consist of extensive sheets of sheared, fine- to coarse-grained hornblende quartz diorite, tonalite, granodiorite, granite and minor trondhjemite, ranging in age from ∼294 to 288 Ma. This intrusive suite is metaluminous and shows a typical calc-alkaline trend. Their NMORB-normalized trace element signature shows depletion of Ta and Nb, and enrichment in Rb, Th and U. Light rare earth elements (LREEs) are enriched and their mid- to heavy rare earth element (MREE-HREE) pattern is flat, suggesting a subduction-related environment with possible crustal contamination. Deformation in the Baidunzi Complex, including intense shearing and folding, is characterized by oblique reverse and rare normal kinematics. The Baidunzi Complex is interpreted to record an ∼294 to 288-Ma Andean-type arc, which may have formed on a transient passive continental margin.

The Ganquan Complex is separated from the Baidunzi Complex by a major shear zone. It mainly comprises felsic volcanic and volcaniclastic sedimentary rocks, and is divided into a lower and upper sequence separated by an unconformity. U-Pb ages and ϵHf values of detrital zircons confirm a genetic relationship between the two sequences. The lower and upper sequences have U-Pb zircon ages of ∼295–286 and ∼283–281 Ma, respectively, whereas the ϵHf values of zircon from volcanic rocks vary from −4.2 to +1.9 and +1.3 to +7.6, respectively, indicating the magma source attained a more juvenile signature over time. Both sequences are geochemically similar, sharing an arc-signature, characterized by LREE enrichment, flat HREE patterns and negative Eu anomalies. Their trace element signatures show enrichment in large-ion lithophile elements (LILEs) and negative Nb, Ta, Sr and Eu anomalies when normalized to the primitive mantle, suggesting a high degree of fractionation and supra-subduction zone (SSZ) affinity. Given their distinctive deformation history and magmatic characteristics, the Ganquan Complex and the Baidunzi Complex, although partially synchronous, are interpreted to represent the two separate arcs. Their subsequent amalgamation is marked by a voluminous ∼281 Ma hornblende gabbro, stitching along the intervening shear zone.

The spatially closely associated Liuyuan Complex, recording active magmatism between ∼290 and 282 Ma, also shows correspondence in age with the Ganquan Complex. There has been intense debate in the literature whether the Liuyuan Complex formed as an arc-related (fore-arc or back-arc) ophiolite [3, 4, 6] or as a continental rift [7]. Detailed mapping and petrological studies allow the Liuyuan Complex to be subdivided into a troctolite, melatroctolite and varitextured olivine gabbro, separated from overlying pillow basalt and chert beds by a newly discovered laterally continuous sheeted dyke complex (Fig. 1c). The basalts show a typical SSZ signature, with positive Th and negative Nd anomalies. The low V/Ti ratios combined with La/Sm and La/Nb similar to Tonga, Izu Bonin and Mariana back-arc basin basalts suggest the Liuyuan Complex formed in a back-arc basin, which is consistent with the presence of coeval arc rocks in the adjacent Ganquan Complex [4]. Combined with stratigraphic and structural studies, it is suggested that the Liuyuan Complex formed during the opening of an oceanic back-arc basin north of the south-facing Ganquan arc (associated with a north-dipping subduction zone) [8]. Meanwhile, progressive increase in juvenile isotope signatures over time in the Ganquan possibly resulted from arc-trench migration, synchronous with the back-arc spreading.

The late Permian-early Triassic Heishankou sedimentary sequence, with fold-and-thrust-style deformation, has characteristics typical of foreland basin deposits. It developed unconformably on rocks of the above complexes and contains a fining-upward formation at the bottom and a syn-kinematic conglomerate formation at the top. The bottom formation comprises a unit of conglomerate with clasts of granitoids and felsic volcanics, possibly derived from the Baidunzi and Ganquan Complex, a cross-bedded sandstone unit, and a siltstone unit with flaser laminations in stratigraphic order. It is unconformably overlain by the conglomerate formation, which is featured by additional presence of mafic pebbles from the Liuyuan Complex as well as fragments of the underlying formations. These indicate that the sequence started deposition syn-tectonically during uplift and exhumation of the Ganquan and Baidunzi Complexes and locally overstepped them. The Heishankou sedimentary sequence is capped by the syn-tectonic ‘conglomerate formation’, which records erosion of the Liuyuan Complex after it had breached sea level, with the basin progressively migrating southward over the accreted and imbricated complexes and underlying units, as shown in the map.

In summary, detailed mapping in the southern Beishan Orogen indicates that the tectonic evolution of the area involves the formation and amalgamation of two distinctive arcs: a north-facing Andean-type continental arc and a south-facing oceanic arc–back-arc system. The accretion/collision events are at least in part recorded in the Heishankou sedimentary sequence. The results suggest that the closure of the Paleo-Asian Ocean involved multi-phase arc accretion/collision events during the Permian.

## References

[1] Windley BF Alexeiev D Xiao WJ et al. J Geol Soc London 2007; 164: 31–47. 10.1144/0016-76492006-022

[2] Xiao WJ Windley BF Yuan C et al. Am J Sci 2009; 309: 221–70. 10.2475/03.2009.02

[3] Xiao WJ Mao QG Windley BF et al. Am J Sci 2010; 310: 1553–94. 10.2475/10.2010.12

[4] Mao Q Xiao W Windley BF et al. Geol Mag 2012; 149: 483–506. 10.1017/S0016756811000811

[5] van Staal CR Hong T Lin S et al. Bedrock Geological Map of the Baidunzi-Heishankou Area, Guazhou, Gansu, China, scale 1:25,000. China Geological Survey, 2021. doi: 10.35080/data.C.2021.P01.

[6] Tian ZH Xiao WJ . Geol J 2020; 55: 2023–43. 10.1002/gj.3700

[7] Wang Y Luo Z Santosh M et al. Geol Mag 2017; 154: 265–85. 10.1017/S0016756815001077

[8] Santos GS Hong T van Staal CR et al. Geol J 2022; doi: 10.1002/gj.4609. 10.1002/gj.4609

